# Characteristic of Gelatine, Carrageenan and Sodium Alginate Hydrosols Treated by Direct Electric Current

**DOI:** 10.3390/polym8080275

**Published:** 2016-07-30

**Authors:** Żaneta Król, Magdalena Malik, Krzysztof Marycz, Andrzej Jarmoluk

**Affiliations:** 1 Department of Animal Products Technology and Quality Management, Wroclaw University of Environmental and Life Sciences, Chelmonskiego 37/41, 51-630 Wroclaw, Poland; andrzej.jarmoluk@up.wroc.pl; 2 Faculty of Chemistry, Wroclaw University of Technology, Smoluchowskiego 23, 50-370 Wroclaw, Poland; magdalena.malik@pwr.edu.pl; 3 Department of Environment Hygiene and Animal Welfare, The Faculty of Biology and Animal Science, Wrocław University of Environmental and Life Sciences, Chelmonskiego 38 C, 50-630 Wroclaw, Poland; krzysztof.marycz@up.wroc.pl

**Keywords:** gelatine, carrageenan, sodium alginate, hydrosol, direct current, rheology, SEM, FTIR, storage

## Abstract

The aim of the study was to investigate the effect of using direct electric current (DC) of 400 mA for five minutes on the physiochemical properties of gelatine (2%, 4%, and 8%), carrageenan (1.5%, 2%, and 2.5%) and sodium alginate (0.75%, 1%, and 1.25%) hydrosols with different sodium chloride concentration. The pH, oxidation-reduction potential (ORP), electrical conductivity (EC), available chlorine concentration (ACC) and rheological parameters were measured. Moreover, Fourier transform infrared spectroscopy (FT-IR) and Scanning Electron Microscopy (SEM) analysis were carried out. The results have shown that pH, ORP, EC and ACC values are changed upon applying DC and the magnitude of change depends on the concentration of the polymer and the addition of sodium chloride. After seven days of storage, the ACC of the samples exposed to DC decreased by 88%–96%. The FT-IR spectra demonstrated that the structure of gelatine, carrageenan and sodium alginate are not significantly affected by DC. Furthermore, the use of DC did not affect the flow and gelation temperature of the hydrosols. These results suggest that the use of DC did not cause undesirable changes in hydrosols layer and these innovative materials can be used, e.g., for food preservation.

## 1. Introduction

Hydrocolloids are hydrophilic polymers that are readily dispersive, fully or partially soluble, and prone to swelling in water. They are widely used in a variety of industrial sectors because of the number of their functions including thickening and gelling aqueous solutions; stabilizing foams, emulsions and dispersions; inhibiting ice and sugar crystal formation; the controlled release of flavors; etc. Moreover, they are biodegradable, non-toxic, biocompatible and bioactive [1]. Consumers have always felt the need for foods with better texture, taste and other organoleptic properties. Currently, consumers tend to choose safe, high quality unprocessed products, which became one of the driving factors that boosted the development of the hydrocolloids market [2]. Nowadays, hydrocolloids are also used as edible and biodegradable coatings or films to control moisture loss, adverse chemical reactions, microbiological stability, etc. [3,4]. Furthermore, natural polymer-based hydrogels have been of great interest to biomaterial scientist, e.g., as wound dressing materials [5].

Polymers can be classified based on a variety of characteristics, including the nature of side groups (neutral or ionic). Natural amphipathic polymers are gelatine, collagen, carboxymethyl chitin and fibrin. The group of cationic polymers includes chitosan and polylysine, whereas anionic polymers include alginic acid, carrageenan, pectin, chondroitin sulfate, and dextran sulfate [6]. This study also evaluates the effects of using electric field on hydrosols. For the purpose of this study, there were three different polymers chosen with the ability to form gels: sodium alginate, carrageenan and gelatine. Sodium alginate is a natural anionic polysaccharide extracted from various species of brown algae. It is composed of 1–4 linked α-l-guluronic (G) and β-d-mannuronic (M) acid residues with free hydroxyl (OH^−^) and carboxylate (–COO^−^) groups distributed along the backbone [7]. Sodium alginate is a pH- and electric field-responsive polymer. Below the pΚ_a_ values of guluronic and mannuronic acid units, 3.38 and, 3.65, respectively, the carboxyl groups are in protaned (–COOH) form while above the p*Κ*_a_ values the groups are in ionized (–COO^−^) form [8]. Carrageenan and gelatine are also sensitive to electrical fields [9]. Carrageenan is an anionic polysaccharide extracted from certain species of red seaweed (*Rhodophyta*) [10]. Chemically, it is a linear polymer, sulfated galactan, composed of alternating disaccharide repeating units of 3-linked β-d-galactopyranose (G units) and 4-linked α-d-galactopyranose (D units) or 4-linked 3,6-anhydro-α-d-galactopyranose (DA units). There are three main types of carrageenan (κ, ι, λ-carrageenans) that differ from one another according to its 3,6-anhydrogalactose content and the number of sulfate groups present in the structure along the chain [11]. Gelatine is a fibrous protein obtained by partial denaturation of collagen. The chemical composition of gelatine is similar to the native collagen, which is composed of three α-chains in the triple helix [12]. These chains provide an ideal geometry for inter-chain hydrogen bonding. Industrial gelatines are mixtures of different compounds: α-chains, β-chains and γ-chains. Gelatine molecule structure is mainly composed of repeating sequence of GLY-X-Y triplets, where X is mostly proline and Y is mostly hydroxyproline [13]. Polymer hydrogels can exhibit phase transition in response to change in external condition such as electric current, pH, ionic strength, temperature, are called “smart” gels. These unique features of hydrogels are the future of drug delivery, cell encapsulation and tissue engineering [14,15,16].

There is much literature on the use of direct electric current (DC) in hydrosol/hydrogel layer in the field of dermal and transdermal drug delivery [17,18,19]. Iontophoresis is a non-invasive technique that has been studied since 1900. It is based on the application of a weak electric current to release drugs through biological membranes [20]. The DC was also used for electrolytic stimulation of bacteria *Enterobacter dissolvens* [21] and acidophilic bacteria [22]. In both cases, the results have shown that weak DC (10–100 mA) inhibits cell growth. The same effect was observed in our previous study [23]. The use of DC (10–30 mA) inhibited the growth of *S. aureus* and *Y. enterocolitica* and the addition of sodium chloride enhanced the effect. Although antimicrobial properties have been confirmed, there are no studies on the application of hydrosols treated with DC as a form of edible food component. The authors suggest that flow of a weak DC in hydrosols may be used as a new food decontamination method. Innovative hydrosols with antibacterial activity can be used in industry, e.g., in the co-extrusion coating process, which is promising method of laminar sanitization. In these studies, we decided to increase the current from 10 to 30 mA to 400 mA to provide presence of available chlorine concentration (ACC) that exhibits antibacterial activity upon storage [24,25]. The change makes the use of DC not only method for non-thermal sterilization but also it allows to extend the shelf life and reduce or eliminate addition of preservatives.

The aim of the study was to evaluate the changes in physiochemical and rheological properties of gelatine, carrageenan and sodium alginate hydrosols after the application of direct electric current (DC).

## 2. Experimental Section

### 2.1. Apparatus

The apparatus used to treat the samples with direct electric current (DC) has been presented in our recent work [26]. The hydrosols were poured into holes in Teflon plate. The graphite electrodes were kept in contact with the opposite surface of the hydrosol. The electric current was provided from a DC power supply, Major Science MP-SAP (Major Science, Saratoga, NY, USA). During all of the experiments, the samples were treated with DC of 400 mA for five minutes. The controls were treated in exactly the same manner as the research sample, except that no electric current was applied.

### 2.2. Materials

Gelatine from porcine skin (180 Bloom) was purchased from Weishardt (Graulhet, France) and κ-carrageenan extracted from *Euchema cottoni* was acquired from Regis (Kraków, Poland). Alginate FD 125 extracted from *Laminaria digitata* (molecular weight 140 kDa, particle size max. 2% > 620 μm, M:G ratio = 1.2) was obtained from Dupont GRINSTED^®^, Grindsted, Denmark.

### 2.3. Preparation of Hydrosols

The Gelatine/Carrageenan/Sodium alginate was dissolved in distilled water containing NaCl. The composition of obtained solutions is shown in Table 1. The gelatine and carrageenan sols were heated to 60 °C and stirred continuously (IKA, RW 20 digital) at 300 rpm for ten minutes, while the sodium alginate sols were stirred for thirty minutes in room temperature. After DC application, polymers solutions were homogenized by homogenizer IKA (T18 basic, Ultra Turrax, Staufen, Germany) for 15 s.

### 2.4. Hydrosols Characterization

#### 2.4.1. Physiochemical Properties

The pH, oxidation-reduction potential (ORP) and electrical conductivity (EC) of hydrosols were measured using a pH/mV/ISE Meter (Seven Multi™ model S40, Mettler Toledo, Warsaw, Poland) equipped with a pH electrode (Inlab Routine Pro, Mettler Toledo), ORP electrode (Inlab Redox Pro, Mettler Toledo) and conductivity electrode (InlabLab 731, Mettler Toledo), respectively. The available chlorine concentration (ACC) was determined by the iodometric method [22]. The pH, ORP, EC and ACC were measured during storage in gelatine, carrageenan and sodium alginate samples (G0.75N0.2, C0.75N0.2, and A0.75N0.2) with lower concentration of polymer to avoid gelling. The samples were stored in open state in the dark at room temperature (20–22 °C) for 7 days.

#### 2.4.2. Rheological Measurements

##### Flow Properties

Flow property was determined using HAAKE RheoStress 6000 rheometer (Thermo Scientific, Karlsruhe, Germany). The experiment was carried out at 25 °C for sodium alginate, 35 °C for gelatine and 45 °C for carrageenan samples in order to get a viscous aqueous solutions. All tests were performed using cone sensor (C60/1° Ti L, Thermo Scientific, Karlsruhe, Germany) and measuring plate (TMP60 Steel 18/8, Thermo Scientific, Karlsruhe, Germany) in CS mode. The measuring device was driven using RheoWin Job Manager version 4.00 software (Haake, Vreden, Germany). The shear stress (τ) and viscosity (η) measurements were obtained under controlled shear rate of 0–100 s^−1^ within 2 min. Shear stress (*τ*_0_$\dot{\gamma }$) and viscosity (*η*_0_$\dot{\gamma }$) at minimum sheer rate (0 s^−1^) and shear stress (*τ*_100_$\dot{\gamma }$) and viscosity (*η*_100_ $\dot{\gamma }$) at maximum sheer rate (100 s^−1^) were measured. The experimental data were also fitted using Ostwald de Waele (Equation (1)) and Herschel–Bulkley models (Equation (2)), as follows:

(1) $$\tau =K{\dot{\gamma }}^{n}$$

(2) $$\tau =\tau_{0}+K{\dot{\gamma }}^{n}$$

where τ is the shear stress (Pa), $\dot{\gamma }$ is the shear rate (s^−1^), *K* is the consistency index (Pa·s^n^), *n* is the flow behavior index (dimensionless), and $\tau_{0}$ is the yield stress (Pa).

##### Gelation and Flow Temperatures

Gelation and flow temperatures were determined using a Haake RheoStress 6000 rheometer (Thermo Scientific, Karlsruhe, Germany) operating in oscillatory mode, with a strain of 5% and frequency of 1 Hz. These conditions were checked to stand in the linear viscoelastic region. Storage modulus *G*′ and loss modulus *G*″ were recorded as a function of temperature. One milliliter of gelatine or carrageenan hydrosols was applied onto the measurement plate. A cone/plate geometry with a cone of 0.52 mm was used and hydrosols evaporation was prevented by the use of paraffin oil around measured area. The results of *G*′ and *G*″ were obtained in two stages: during cooling from 75 °C to 15 °C and during heating from 15 °C to 75 °C. Temperature ramps of ± 1.2 °C·min^−1^ were applied. Determination of the equilibrium *G*′ = *G*″ for evaluation of variability of both modules as a function of temperature specifies the conditions of sol-gel phase transition (stage I) and gel-sol (stage II). The gelation (*T*_g)_ and flow (*T*_f_) temperatures of hydrosols were obtained from the point of intersection of curves. All tests were performed using cone sensor (C60/1° Ti L, Thermo Scientific, Karlsruhe, Germany) and measuring plate (TMP60 Steel 18/8 Thermo Scientific, Karlsruhe, Germany) in CS mode. The measuring device was running on RheoWin Job Manager version 4.00 software (Haake, Vreden, Germany).

#### 2.4.3. Fourier Transform Infrared Spectroscopy

The spectral measurements were performed in The Laboratory of Vibrational Spectroscopy belonging to The Faculty of Chemistry at Wrocław University of Technology (Wrocław, Poland). The middle-infrared spectra (4000–400 cm^−1^) were collected on a Fourier transform, Bruker VERTEX 70 V vacuum spectrometer (Bruker Optik GmbH, Ettlingen, Germany) equipped with an air-cooled DTGS detector. The gelatine, carrageenan and sodium alginate samples were placed on the diamond crystal of the Attenuated Total Reflection accessory. The spectral data were recorded at the resolution of 2 cm^−1^ with collected of 64 scans and further elaborated using Bruker OPUS software (Bruker Optik GmbH, Ettlingen, Germany).

#### 2.4.4. Scanning Electron Microscopy

Scanning Electron Microscopy (SEM) of the gelatin, carrageenan and sodium alginate hydrosols microstructure were evaluated using the EVO LS15 ZEISS Scanning Electron Microscope (Zeiss, Jena, Germany). The investigated samples were prepared as previously described by Śmieszek et al. [27]. The procedure includes fixation in 0.5% cacodyl buffer (pH = 4), dehydration in rising alcohol gradient (form 50% till 100%) and dry using critical point dying technique (CPD, SPI-DRY Critical Point Dryer, UK). Next, the dehydrated hydrosols were sputtered with gold for 150 s using a sputter coater Scancoat 6 (Edwards, London, UK), which finally generated a 10 nm thick gold layer. Each coated sample was examined using a voltage of 20 kV.

### 2.5. Statistical Analysis

Each experiment was performed in triplicate. The effect of three independent categorical variables, such as the current, polymers concentration and sodium chloride concentration, were evaluated. A statistical analysis was performed with univariate and multivariate analysis of variance (ANOVA) using Statistica 10 (StatSoft, Cracow, Poland). The differences between the mean values were identified by the Duncan Test with a confidence level at *p* < 0.05.

## 3. Results and Discussion

### 3.1. Physiochemical Properties of Hydrosols

The application of DC in hydrosols layer caused the chemical reaction at the electrodes according to Equations (3)–(7) [28]:

**Anode**:  2H_2_O → 4H^+^ + O_2_ ↑ + 4e^−^, (3)

2NaCl → Cl_2_ ↑ + 2e^−^ + 2Na^+^, (4)

Cl_2_ + H_2_O → HCl + HOCl, (5)

**Cathode**:  2H_2_O + 2e^−^ → 2OH^−^ + H_2_ ↑, (6)

2NaCl + 2OH^−^ → 2NaOH + Cl^−^ (7)

During electrolysis, the negatively charged ions moved to the anode and loose electrons and form oxygen gas, chlorine gas, hypochlorite ion, hypochlorous acid and hydrochloric acid. Positively charged ions, including hydrogen and sodium, moved to the cathode to take up electrons and form hydrogen gas and sodium hydroxide, respectively [29].

**Figure 1:**
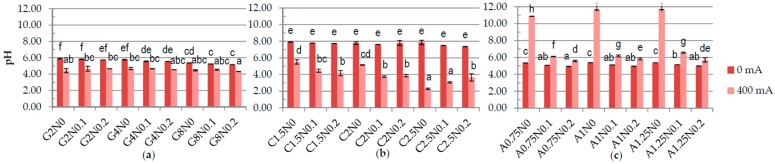
The effects of using direct electric current (DC) on the pH of (**a**) gelatine; (**b**) carrageenan and (**c**) sodium alginate hydrosols with different polymer and NaCl concentration. ^a–i^ Different letters indicate significantly different groups determined by Duncan’s test (*p* < 0.05).

**Figure 2:**
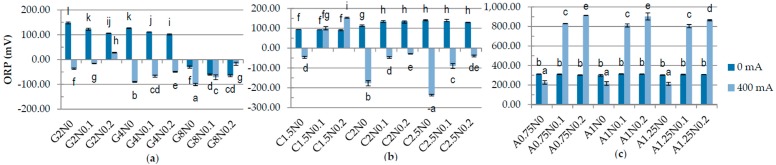
The effects of using DC on the oxidation-reduction potential (ORP) of: (**a**) gelatine; (**b**) carrageenan; and (**c**) sodium alginate hydrosols with different polymer and NaCl concentration. ^a–l^ Different letters indicate significantly different groups determined by Duncan’s test (*p* < 0.05).

**Figure 3:**
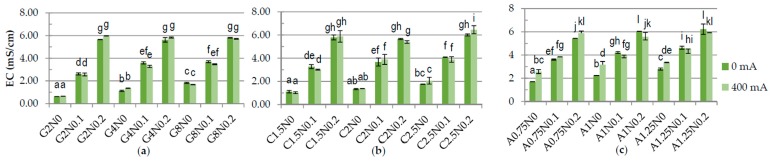
The effects of using DC on the electrical conductivity (EC): of (**a**) gelatine; (**b**) carrageenan; and (**c**) sodium alginate hydrosols with different polymer and NaCl concentration. ^a–l^ Different letters indicate significantly different groups determined by Duncan’s test (*p* < 0.05).

**Figure 4:**
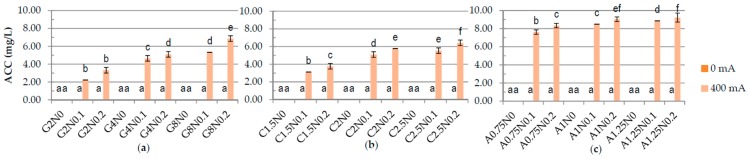
The effects of using DC on the available chlorine concentration (ACC) of: (**a**) gelatine; (**b**) carrageenan; and (**c**) sodium alginate hydrosols with different polymer and NaCl concentration. ^a–f^ Different letters indicate significantly different groups determined by Duncan’s test (*p* < 0.05).

One of the results of carrying out electrolysis is the splitting of water according to Equations (3) and (6), causing changes in pH [28]. The results have shown significant difference in the pH between samples treated and not treated with DC (Figure 1). The pH of gelatine and carrageenan samples was lower after application of DC. The lowest pH was measured for the 2.5% carrageenan samples with 0% and 0.1% of sodium chloride (2.27 and 3.06, respectively) and for the G8N0.2 gelatine sample (4.33) treated with DC. The pH of sodium alginate samples was higher after application of DC. The highest pH was obtained for sodium alginate variants treated with DC and without NaCl while the lowest for samples with 0.2% of sodium chloride. These results may be explained by the fact that during electrolysis on the anode the gel is formed. The parameters of formed gel are nearly equivalent to the parameters of the acidic electrolyzed water AEW (pH of 2–3, ORP > 1100 mV, ACC of 10–90 ppm), while the rest of the solution has pH of 10–13 and ORP of −800 to −900 mV, as alkaline electrolyzed water [28,29]. The higher the addition of sodium chloride the weaker the gel becomes and more easily it disintegrates during the homogenization. There were significant differences in the ORP in the samples depending on the concentration of polymers and application of DC (Figure 2). The lowest ORP in gelatine, carrageenan and sodium alginate samples was measured for the variants treated with DC with the highest concentration of polymer and without NaCl, −100.46 mV, −237.2 mV, and 212.5–226.1 mV, respectively. The same dependency for carrageenan samples was observed by Brychcy et al. [30]. EC and ACC concentration strictly depends on NaCl values [29,31,32]. The highest values of EC (Figure 3) and ACC (Figure 4) were measured in samples with 0.2% NaCl. Chlorine and chlorine-containing compounds due to their efficacy, availability and relative low cost have been the most commonly used sanitizers in food processing [25]. Cao et al. [24] demonstrated that slightly acidic electrolyzed water (SAEW) with an available chlorine concentration of 2 mg/L reduced *S. enteritidis* about 1.00 log. At a pH of 5.0–6.5, the effective form of chlorine compounds in SAEW is almost the hypochlorous acid (HOCl), which has strong antimicrobial activity [33]. Despite the fact that HOCl exerts potential cytotoxicity [34], chemical oxidants are commonly used, e.g., in water treatment process [35]. According to Izumi [36] and Mokudai et al. [37], optimized electric field conditions and addition of sodium chloride allow to avoid toxicity effect to normal cells while antibacterial effect is still obtained. Brychcy et al. [30] demonstrated that carrageenan and gelatine hydrosols incorporated with acid electrolyzed water (AEW) with ACC concentration of 8.20 mg/L and 2.19 mg/L, respectively, have antibacterial activity. The highest reduction of *Staphylococcus aureus* and *Escherichia coli* was observed after treatment with carrageenan (1.80 and 1.59 log reduction, respectively) and gelatine (2.10 and 1.56 log reduction, respectively) hydrosols incorporated with 0.1% of electrolyzed sodium chloride solution exposed to electrolysis for ten minutes.

**Figure 5:**
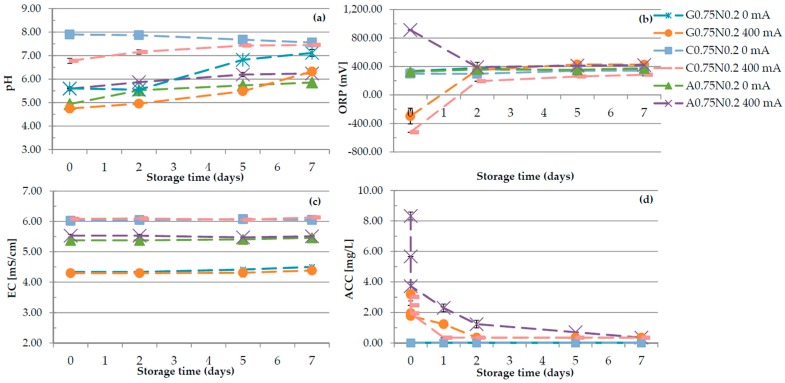
The effects of using DC on: (**a**) the pH; (**b**) the ORP; (**c**) the EC; and (**d**) the ACC of gelatine (G0.75N0.2), carrageenan (C0.75N0.2) and sodium alginate (A0.75N0.2) hydrosols measured during seven days of storage.

The EC of all samples remained constant during storage, whereas pH, ORP, and ACC changed with storage time (Figure 5). The pH of most of all samples increased during storage. The highest increase of pH from 5.61 to 7.12 and from 4.75 to 6.33, was observed for G0.75N0.2 0 mA and G0.75N0.2 400 mA variant, respectively. The differences in the pH of the gelatine gels during storage were also reported by Król and Jarmoluk [23]. There were no significant differences in the ORP before and after seven days of storage in the variants not treated with DC. After two days of storage, the ORP of gelatine, carrageenan and sodium alginate samples treated with 400 mA changed to the values approximating the value reading for samples not treated with DC. In the second day of storage, the ORP values decreased from 913.75 to 391.7 for A0.75N0.2 400 mA variant. The higher ORP values were noticed for G0.75N0.2 and for C0.75N0.2 treated with DC and it increased from −296.65 mV to 391.70 mV for gelatine and from −523.75 mV to 193.80 mV for carrageenan sample. A similar result has been observed by Cui et al. [31] for the change of ORP values in water. The authors suggest that increase and reaching an equilibrium ORP value around 750 mV can occur because the ORP values of electrolyzed water samples below 750 mV have reducing power and tend to react with oxidized species. The oxidation of low ORP water leads to increase in ORP. These may proceed slowly because water does not contain many reducing species before and after electrolysis. Figure 5d shows that ACC of all samples greatly decreased with storage time. There was 96% decrease in ACC of sodium alginate samples after seven days of storage. However, ACC of gelatine and carrageenan decreased by approximately 89% and 88%, respectively, after two days, but reached level maintained during storage. Cui et al. [31] concluded that electrolyzed water stored in open condition had greater loss of ACC compared to storage in closed condition. According to Len et al. [38], under open condition, the loss of chlorine by evaporation in AEW followed the first-order kinetics, which is not the case under closed conditions.

### 3.2. Rheological Measurements

#### 3.2.1. Flow Properties

The results of linear oscillatory shear measurement and solution viscosity are presented in Table 2. The Ostwald de Waele model and Herschel–Bukley model satisfactorily fitted experimental data (*R*^2^ > 0.926). The three types of fluids can be identified on the basis of the value of their flow behavior index (*n*): Newtonian fluid *n* = 1, shear thinning fluid *n* < 1 and shear thickening or dilatant fluid *n* > 1. All tested hydrosols showed pseudo plastic behavior (*n* < 1). According to Junyi et al. [39], sodium alginate hydrosols with concentration range from 0.125% to 1.5% (*w*/*v*) and temperature from 5 to 35 °C exhibit non-Newtonian shear-thinning behavior. The apparent viscosity (η) strictly depended on polymer concentration, which is in agreement with Diggirala and Deluca’s statement [40]. Moreover, the apparent viscosity was highly dependent on the shear rate ($\dot{\gamma }$) at which shear stress was measured. The highest viscosity at maximum $\dot{\gamma }$ was noticed for G2N0, C2.5N0.2 and A1.25N0 not treated with DC and was equal to 17.00, 471.10 and 239.60, respectively. After DC was applied, the apparent viscosity of carrageenan and sodium alginate significantly decreased. For G8N0, C2.5N0 and A1.25N0 variants, viscosity decreased from 17.00 down to 1.53, from 471.10 down to 179.28 and from 239.60 down to 179.45, respectively. The results have shown (Table 2) that after applying DC not only viscosity decreased but all measured parameters. The results of the changes in flow properties of sodium alginate may be explained by the fact that during electrolysis on the anode the gel is formed. Alginate solution can form gels by lowering the pH below the pKa value of the guluronic residue (pH < 3.0) [41]. After applying DC the gel with the rest solution was homogenized, but the strength of the gel was large enough that it could not be completely destroyed. The lower viscosity of carrageenan samples treated with DC could be caused by the small partial hydrolysis of the polysaccharide, which can occur at low pH [42]. As mentioned in Section 3.1, during the electrolysis, pH near the anode is much lower and near the cathode it is much higher than pH of mixed solution after DC treatment. Venegas-Sanchez et al. [43] treated carrageenan hydrosols with ultrasound (US) and obtained solutions tested for viscosity. They found out that after US treatment the viscosity of treated samples decreased. The authors suggest that the reason of viscosity reduction was the condition of the polymer coil conformation, which was expanded or shrunk by electrostatic repulsion of the SO_3_^−^ groups. Moreover, they noticed that presence of NaCl in aqueous solution caused a greater decrease of viscosity, which is in agreement with our results. The salt can shield the electrostatic repulsion of SO_3_^−^ groups of the carrageenan segments and to expand the polymer chains. According to Pang et al. [44], we assumed that changes in polymer conformation of gelatine after applying DC were also the reason of decrease of all the measured parameters. Different hydrosols have different characteristic curves due to different relation between viscosity and shear rate [45]. The results have shown that the shear stress increased with the increase of polymers concentration. The highest shear stress was exhibited by G8N0, C2.5N0 and C1.25N0 variant. This could be due to increase in the intermolecular interactions between the polymer’s molecules [39]. Moreover, it was observed that consistency coefficient (*k*) increased with the concentration of polymers. A similar result was obtained by Gomez-Diaz and Navaza [46]. Yield stress can be defined as the force which fluid must be exposed to in order to start flowing. This parameter gives the information about the resistance of the fluid structure to deformation or breakdown [47]. The lowest *τ*_0_ was noticed for G2N0, C1.5N0.2 and A0.75N0 variants.

#### 3.2.2. Gelation and Flow Temperatures

**Figure 6:**
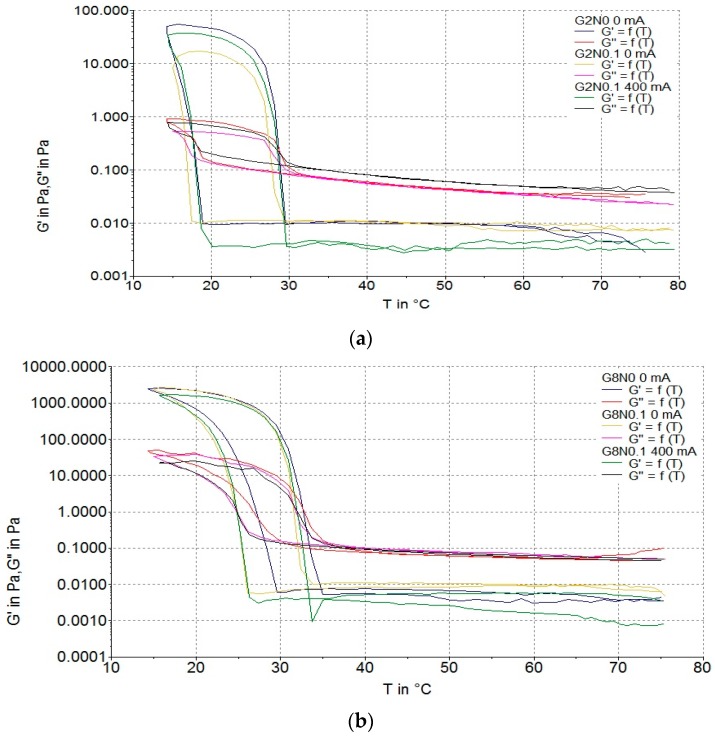
Storage and loss modulus of samples: (**a**) G2N0 0 mA, G2N0.1 0 mA, and G2N0.1 400 mA; and (**b**) G8N0 0 mA, G8N0.1 0 mA, and G8N0.1 400 mA as a function of time.

**Figure 7:**
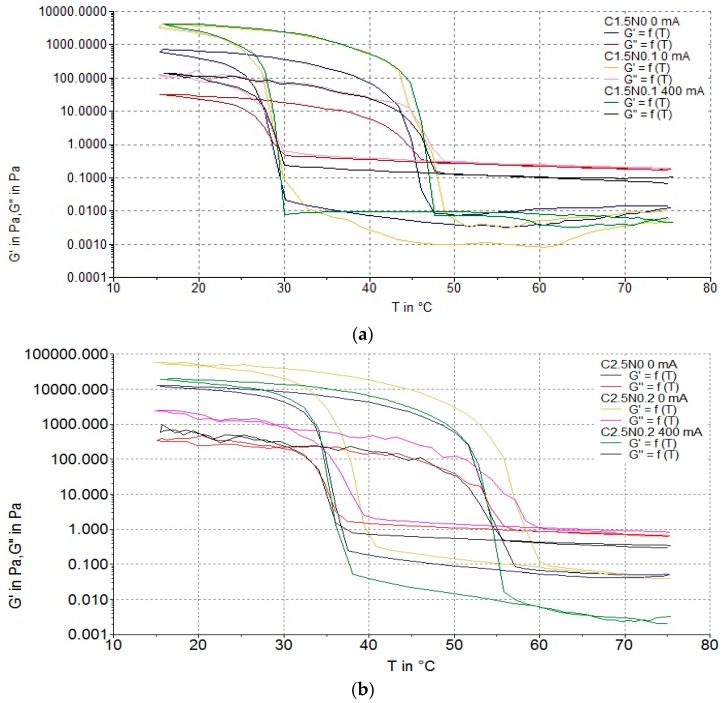
Storage and loss modulus of samples: (**a**) C1.5N0 0 mA, C1.5N0.1 0 mA, and C1.5N0.1 400 mA; and (**b**) C2.5N0 0 mA, C2.5N0.1 0 mA, and C2.5N0.1 400 mA as a function of time.

Figure 6 and Figure 7 show representative rheological results for gelatine and carrageenan hydrosols. There were no significant differences between the gelation temperature (*T*_g_) in the gelatine hydrosols treated and not treated with DC. The lowest *T*_g_ was measured for samples with 2.0% gelatine (17.2–17.4 °C), while the highest was observed for G8N0.1 variant (27.4 °C). Similar results were observed for carrageenan samples. The lowest *T*_g_ was measured for C1.5N0 variant not treated with DC (28.2 °C) and the highest *T*_g_ was observed for C2.5N0.2 sample not treated with DC (39.0 °C). These results indicate that the higher the concentration of polymer, the higher the *T*_g_ of the hydrosols. This theory is confirmed by Pang et al. [44]. In their study, *T*_g_ of the gelatine gel ranged from 15 to 18 °C at 2.5% to 20–22 °C at 5.0% concentration. In accordance with Brychcy et al. [30], we observed that increasing of NaCl concentration causes higher *T*_g_ of carrageenan hydrosols. Increasing the addition of sodium chloride concentration decreased the electrostatic interaction allowing the triple helix constituents of the gel to freely reorganize in the medium to maximize the entropy which affects the *T*_g_ of the hydrosols [48].

Flow temperature (*T*_f_) of all samples was significantly affected by polymer and NaCl concentration while there was no impact of DC on *T*_f_. The highest (32.6 °C) and the lowest (27.5 °C) values of *T*_f_, of gelatine hydrosols were measured for G8N0.1 and G2N0.1 variants, both not treated with DC. The highest *T*_f_ (57.2 °C) of carrageenan hydrosols was observed for C2.5N0.2 variant with no DC treatment. The *T*_f_ depends on polymer concentration and this result is in agreement with other authors [44,49,50]. An increased concentration of polymer leads to shorter distances between gelatine coils and formation of junction zones, and because of that a higher temperature is needed to destroy the structure [28,44].

### 3.3. Fourier Transform Infrared Spectroscopy

**Figure 8:**
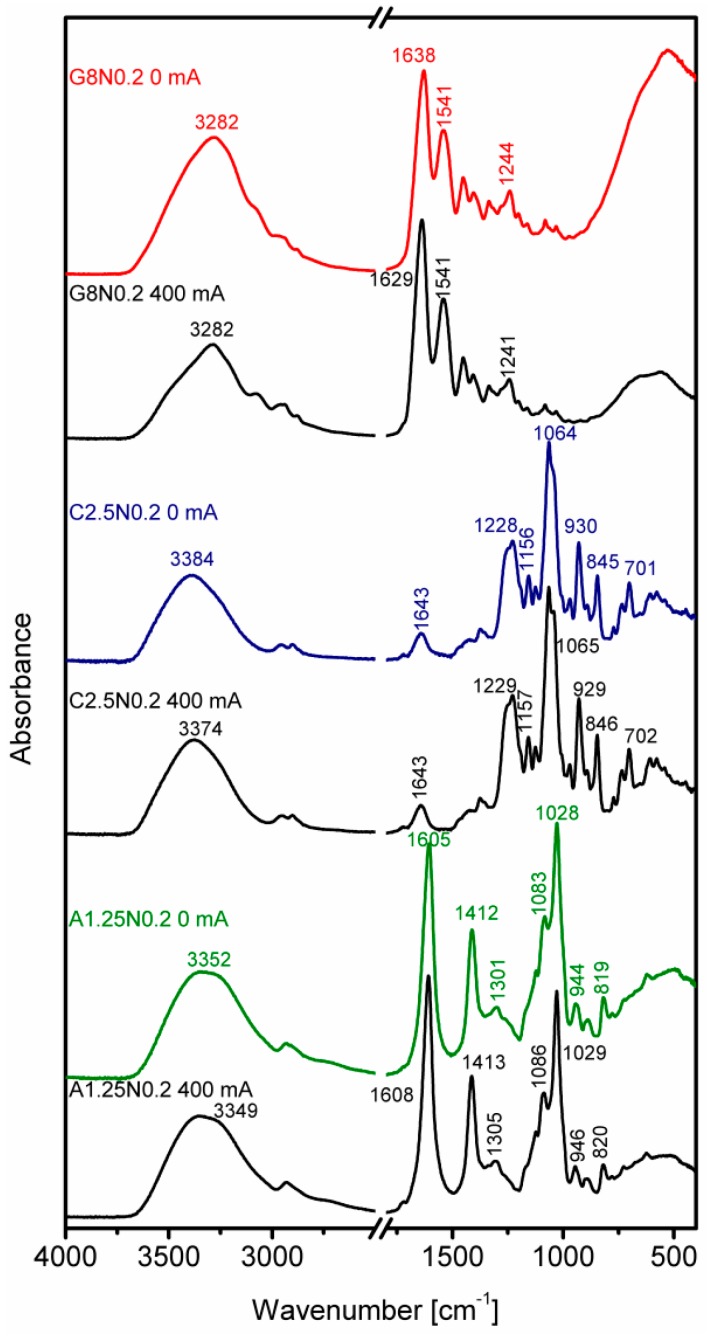
The FT-IR spectra of gelatine (G8N0.2), carrageenan (C2.5N0.2) and sodium alginate (A1.25N0.2) in two variants: control sample (0 mA) and after electrolysis (400 mA). Only discussed bands are labeled.

The main reason for the reported spectroscopic research is the detection of any possible changes in the structures of gelatine, carrageenan and sodium alginate after applying DC. The results are shown in Figure 8 where six IR spectra of respective natural polymers are compared in the following order: gelatine (at the top), carrageenan (in the middle) and sodium alginate (at the bottom). For each polymer, two different spectra are presented, one of them is for the control sample (e.g., G8N0.2 0 mA) and the other one is the spectrum of the same polymer after the electrolysis treatment (e.g., G8N0.2 400 mA).

While discussing the spectroscopic properties of gelatine, the Fourier transform infrared spectroscopy (FT-IR) focuses mainly on peptide bands named Amide A and Amide I–III. In the present case, the Amide A band, resulting from intense IR absorptions of N–H stretching vibrations, is observed as broad band centered at about 3282 cm^−1^ (Figure 8). The Amide I band observed for the control sample at 1638 cm^−1^ is mainly associated with the C=O group stretching vibration. This band is 9 cm^−1^ shifted toward lower wavenumbers for the gelatine hydrosol after electrolysis. The Amide II band results mainly from the N–H bending vibration and from small contribution of the C–N stretching vibration. This band is observed for both gelatine samples at 1541 cm^−1^. In the Amide III vibration, the C–N stretching mode is the main component accompanied by the N–H bending and some contributions of backbone and side-chain vibrations [51,52,53]. In present study, this band is observed as the weak peak at 1244 or 1241 cm^−1^. In general, the spectra of both gelatine samples are very similar to each other, which indicates that the structure of gelatine hydrosol is not significantly affected by electrolysis process and its chlorine products.

The next biopolymer investigated by IR spectroscopy is carrageenan. In this case, the analysis of the middle FT-IR spectra is focused on the fingerprint region (1225–700 cm^−1^). Most of the bands observed there are specific for polysaccharides, but there are also bands resulting from the sulfate group vibrations. The highest wavenumber band associated with the asymmetric O=S=O vibration is observed at 1228 (1229) cm^−1^. The most intensive band at 1064 (1065) cm^−1^ is due to the combination of C–O and S=O modes. The absorption at 845 (846) cm^−1^ results from vibrations of C–O–S linkage and it is used as a marker of the carrageenan conformation. Its 845 cm^−1^ position suggests the *kappa*-conformer [54]. Two remaining medium intensity bands at about 930 and 700 cm^−1^ are mainly attributed to vibrations of the C–O–C bridges typical for polysaccharides [54,55].

While discussing the FT-IR spectra of sodium alginate (a typical marine bio-polymer), two spectral regions are usually considered. The first is between 4000 and 2700 cm^−1^ and composes mainly of O–H and C–H stretching vibrations. In the second region, two intense bands at 1605 (1608) and 1412 (1413) cm^−1^ are mainly due to asymmetric and symmetric stretching vibrations of carboxylate group [55]. The contribution of C–OH deformation vibration is also possible for the latter vibration. The second strong band at 1028 (1029) cm^−1^ is mainly associated with the C–O stretching vibrations. Among weak bands, these in 950–750 cm^−1^ “fingerprint” region are frequently analyzed for carbohydrates. The band at 944 (946) cm^−1^ is assigned to the C–O stretching vibration of uronic acid residues, whereas the band at 819 (820) cm^−1^ is characteristic for sodium alginate and assigned to mannuronic acids residues [56,57,58]. Weak band at 1301 (1305) cm^−1^ is usually assigned to C–C–H and O–C–H bending vibrations.

As for previous polymers, the IR spectra of both sodium alginate samples are also very similar and bear testimony to the structure preservation. 

As shown in Figure 8, the spectral similarities of the respective samples confirm that the electrolysis as well as its products do not change the structures of gelatine, carrageenan and sodium alginate.

### 3.4. Scanning Electron Microscopy

**Figure 9:**
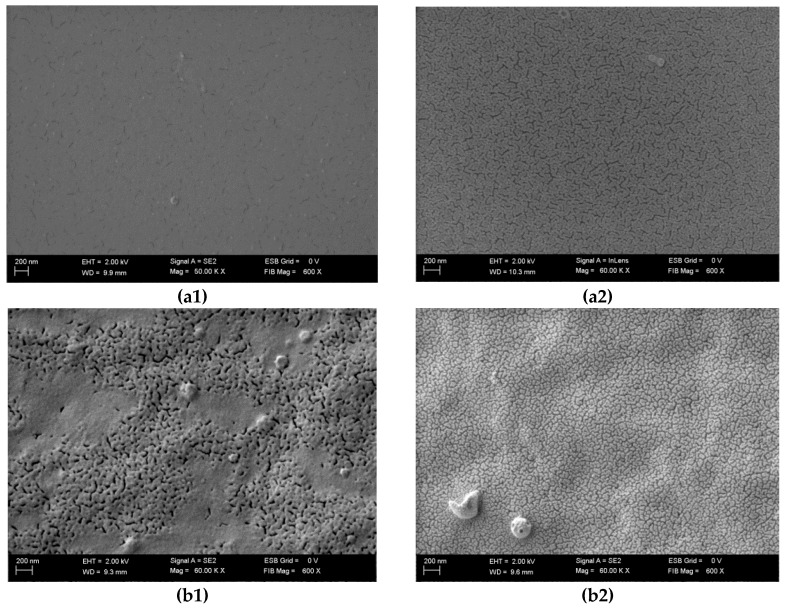

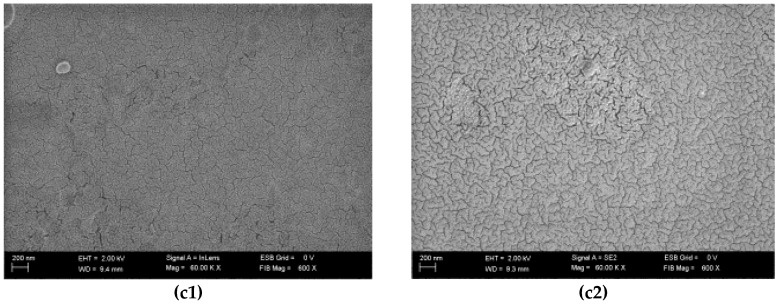
SEM images of the surface of hydrosols of: gelatine (**a1**) G8N0.2, 0 mA, (**a2**) G8N0.2, 400 mA; carrageenan (**b1**) C2.5N0.2, 0 mA, (**b2**) C2.5N0.2, 400 mA; and sodium alginate (**c1**) A1.25N0.2, 0 mA, (**c2**) A1.25N0.2, 400 mA.

SEM results present that application of DC causes morphological changes in treated samples (Figure 9). After electrolysis, the microstructure of the (Figure 9a) gelatine sample is revealed. Moreover, in the (Figure 9b) carrageenan and (Figure 9c) sodium alginate samples treated with 400 mA, more regular microstructures were observed. Hsu and Block [59] and Ramanathan and Block [60] demonstrated that DC contributes to changes in the gel composition and rheological behavior. After applying DC, the charge density and the electrostatic repulsion in hydrosol solutions are changed [61]. Furthermore, changes may occur depending on the following factors: solvent polarity, pH and ionic strength. These factors can be influenced by the extent of ionization of the side chains attached to the polymer backbone [62]. After applying DC, lower pH was observed for all gelatine and carrageenan samples compared to control samples (Section 3.1). At pH below 4.0, solutions with strong electrostatic forces were revealed in gelatine structure [44]. Furthermore, the reason for the differences between sodium alginate samples may arise from formation of gel on the anode during electrolysis and the homogenization process. Król et al. [26] performed mechanical analysis on the three types of gels: control samples (C), gels prepared on the basis of hydrosols treated with DC (400 mA during 5 min) (H), and gels treated with DC (G). The results revealed differences between some parameters of C, H, G gels and the differences were also observed in SEM spectra.

## 4. Conclusions

Physicochemical properties of gelatine, carrageenan and sodium alginate hydrosols with different polymer and sodium chloride concentration after applying DC of 400 mA for five minutes were investigated. Furthermore, the changes in pH, ORP, EC and ACC were measured during storage. The results have shown that measured parameters strictly depend on the type of polymer and its concentration as well as the addition of sodium chloride. After applying DC, the available chlorine generated antibacterial activity, which is confirmed by many researches. The results of FT-IR and flow and gelling temperatures analyses showed that DC did not cause undesirable changes in hydrosols layer. We assumed the use of DC in hydrosol layer provides new properties to these materials that enhance their applicability, e.g., in food industry in the co-extrusion coating process, as a promising method of laminar sanitization.

## Figures and Tables

**Table 1:** The composition of hydrosols.

Run code letters			Gelatine (G) (%)	Carrageenan (C) (%)	Sodium alginate (A) (%)	NaCl (N) (%)
G2N0	C1.5N0	A0.75N0	2.0	1.5	0.75	0.0
G2N0.1	C1.5N0.1	A0.75N0.1				0.1
G2N0.2	C1.5N0.2	A0.75N0.2				0.2
G4N0	C2N0	A1N0	4.0	2.0	1.0	0.0
G4N0.1	C2N0.1	A1N0.1				0.1
G4N0.2	C2N0.2	A1N0.2				0.2
G8N0	C2.5N0	A1.25N0	8.0	2.5	1.25	0.0
G8N0.1	C2.5N0.1	A1.25N0.1				0.1
G8N0.2	C2.5N0.2	A1.25N0.2				0.2

**Table 2:** Flow properties of hydrosols.

Variants	Current (mA)	Ostwald de waele model		Herschel–Bulkley model	Shear stress *τ*_0_$\dot{\gamma }$ (Pa)	Shear stress *τ*_100_$\dot{\gamma }$ (Pa)	Apparent viscosity *η*_0_$\dot{\gamma }$ (mPa·s)	Apparent viscosity *η*_100_ $\dot{\gamma }$ (mPa·s)
		Consistency index *k* (Pa·s)	Flow behavior index *n* (-)	Yield stress *τ*_0_ (Pa)				
**Gelatine**								
G2N0	0	0.009 ± 0.001 ^b,c^	0.749 ± 0.05 ^a,b^	0.026 ± 0.004 ^b^	0.011 ± 0.001 ^a^	0.340 ± 0.007 ^c^	29.90 ± 0.42 ^f^	7.90 ± 0.07 ^e^
G2N0	400	0.015 ± 0.001 ^d,e^	0.688 ± 0.03 ^a,b^	0.004 ± 0.000 ^a^	0.008 ± 0.000 ^a^	0.329 ± 0.007 ^c^	9.33 ± 0.09 ^b,c^	3.24 ± 0.16 ^c^
G2N0.2	0	0.007 ± 0.001 ^a,b^	0.837 ± 0.00 ^b^	0.024 ± 0.002 ^b^	0.012 ± 0.001 ^a^	0.326 ± 0.013 ^c^	9.84 ± 0.11 ^b,c^	3.16 ± 0.01 ^c^
G2N0.2	400	0.008 ± 0.000 ^b,c^	0.844 ± 0.06 ^b^	0.021 ± 0.002 ^b^	0.010 ± 0.000 ^a^	0.346 ± 0.007 ^c^	8.80 ± 0.17 ^b^	3.37 ± 0.06 ^c^
G8N0	0	0.010 ± 0.001 ^c^	0.563 ± 0.23 ^a^	0.023 ± 0.000 ^c^	0.285 ± 0.330 ^a^	1.689 ± 0.017 ^e^	47.55 ± 0.35 ^g^	17.00 ± 0.28 ^f^
G8N0	400	0.014 ± 0.001 ^d^	0.557 ± 0.05 ^a^	0.035 ± 0.002 ^d^	0.027 ± 0.018 ^a^	0.191 ± 0.058 ^b^	13.09 ± 0.16 ^d^	1.53 ± 0.04 ^b^
G8N0.2	0	0.017 ± 0.003 ^e^	0.336 ± 0.08 ^c^	0.003 ± 0.000 ^a^	0.026 ± 0.000 ^a^	0.589 ± 0.010 ^d^	23.45 ± 0.35 ^e^	5.89 ± 0.10 ^d^
G8N0.2	400	0.004 ± 0.000 ^a^	0.796 ± 0.02 ^b^	0.013 ± 0.000 ^b^	0.002 ± 0.000 ^a^	0.131 ± 0.016 ^a^	6.83 ± 0.06 ^a^	1.23 ± 0.04 ^a^
**Carrageenan**								
C1.5N0	0	0.226 ± 0.019 ^a,b^	0.869 ± 0.02 ^a,b^	0.033 ± 0.000 ^a^	0.258 ± 0.011 ^b,c^	15.025 ± 1.407 ^c^	310.50 ± 0.71 ^f^	140.27 ± 0.38 ^e^
C1.5N0	400	0.128 ± 0.003 ^a,b^	0.905 ± 0.01 ^b–d^	0.022 ± 0.001 ^a^	0.129 ± 0.006 ^a^	8.281 ± 0.064 ^a^	129.60 ± 0.71 ^b^	83.60 ± 0.42 ^a^
C1.5N0.2	0	0.164 ± 0.006 ^a,b^	0.925 ± 0.01 ^c,d^	0.218 ± 0.308 ^a^	0.238 ± 0.016 ^b^	11.110 ± 0.735 ^b^	200.61 ± 0.86 ^c^	120.15 ± 0.21 ^d^
C1.5N0.2	400	0.113 ± 0.001 ^a^	0.950 ± 0.00 ^d^	0.015 ± 0.000 ^a^	0.117 ± 0.006 ^a^	8.694 ± 0.103 _a_	119.75 ± 0.35 ^a^	88.58 ± 1.24 ^b^
C2.5N0	0	1.088 ± 0.258 ^c^	0.828 ± 0.05 ^b^	1.416 ± 0.143 ^b^	1.500 ± 0.028 ^f^	47.288 ± 2.850 ^e^	1,587.70 ± 4.95 ^h^	471.10 ± 1.98 ^g^
C2.5N0	400	0.409 ± 0.013 ^b^	0.828 ± 0.01 ^b^	0.089 ± 0.004 ^a^	0.304 ± 0.002 ^d^	8.400 ± 0.028 ^a^	279.30 ± 0.99 ^e^	179.28 ± 1.03 ^f^
C2.5N0.2	0	1.135 ± 0.191 ^c^	0.777 ± 0.03 ^a^	2.350 ± 0.049 ^c^	1.064 ± 0.034 ^e^	38.385 ± 0.990 ^d^	390.10 ± 7.07 ^g^	120.00 ± 1.70 ^d^
C2.5N0.2	400	0.193 ± 0.008 ^a,b^	0.895 ± 0.01 ^b,c^	0.276 ± 0.010 ^a^	0.279 ± 0.045 ^c^	11.080 ± 0.813 ^b^	259.65 ± 0.49 ^d^	110.20 ± 0.28 ^c^
**Sodium alginate**								
A0.75N0	0	0.095 ± 0.000 ^c^	0.966 ± 0.002 ^g^	0.127 ± 0.002 ^d^	0.130 ± 0.001 ^c^	8.104 ± 0.027 ^b^	119.75 ± 0.35 ^d^	80.31 ± 0.27 ^d^
A0.75N0	400	0.131 ± 0.002 ^d^	0.877 ± 0.000 ^a^	0.023 ± 0.000 ^a^	0.121 ± 0.010b ^c^	7.444 ± 0.112 ^b^	110.51 ± 0.71 ^c^	73.35 ± 0.35 ^c^
A0.75N0.2	0	0.080 ± 0.001 ^b^	0.947 ± 0.002 ^f^	0.113 ± 0.015 ^c^	0.108 ± 0.008 ^b^	6.284 ± 0.115 ^a^	93.80 ± 0.99 ^b^	62.15 ± 0.21 ^b^
A0.75N0.2	400	0.054 ± 0.002 ^a^	0.978 ± 0.003 ^h^	0.076 ± 0.002 ^b^	0.086 ± 0.012 ^a^	5.622 ± 0.837 ^a^	71.15 ± 0.07 ^a^	49.95 ± 0.49 ^a^
A1.25N0	0	0.351 ± 0.013 ^h^	0.925 ± 0.000 ^c^	0.435 ± 0.001 ^e^	0.444 ± 0.012 ^g^	24.590 ± 0.608 ^e^	421.10 ± 1.56 ^h^	239.60 ± 0.57 ^h^
A1.25N0	400	0.196 ± 0.004 ^e^	0.930 ± 0.001^d^	0.032 ± 0.000 ^a^	0.295 ± 0.005 ^e^	17.956 ± 0.517 ^c^	260.60 ± 0.85 ^f^	179.45 ± 0.78 ^f^
A1.25N0.2	0	0.336 ± 0.001 ^g^	0.911 ± 0.000 ^b^	0.071 ± 0.000 ^b^	0.405 ± 0.005 ^f^	22.420 ± 0.042 ^d^	370.10 ± 0.14 ^g^	220.05 ± 0.07 ^g^
A1.25N0.2	400	0.223 ± 0.000 ^f^	0.936 ± 0.001 ^e^	0.031 ± 0.000 ^a^	0.259 ± 0.011 ^d^	16.956 ± 0.418 ^c^	240.21 ± 0.30 ^e^	170.60 ± 0.85 ^e^

^a–h^ Values with different letters within the same column differ significantly (*p* < 0.05).
